# Editorial: The ABCDEF bundle: Laying the foundations for long term wellness in ICU survivors

**DOI:** 10.3389/fmed.2022.1056056

**Published:** 2022-10-21

**Authors:** Maria Vargas, Pasquale Buonanno, Katarzyna Kotfis, Annachiara Marra

**Affiliations:** ^1^Department of Neurosciences, Reproductive and Odontostomatological Sciences, University of Naples “Federico II”, Naples, Italy; ^2^Department of Anesthesiology, Intensive Therapy and Acute Intoxications, Pomeranian Medical University, Szczecin, Poland

**Keywords:** post intensive care syndrome (PICS), ABCDEF bundle, communication, family, sedation administration

This Research Topic entitled “*The ABCDEF Bundle: Laying the Foundations for Long Term Wellness in ICU Survivors?*” involved authors from different specializations and numerous countries, confirming the dissemination and implementation of the ABCDEF bundle is a very timely topic worldwide.

Long-term morbidity, with cognitive, physical, and emotional symptoms, is common in critical illness survivors, with significant consequences for patients' and caregivers' quality of life. Consequently, health provider, families, and researchers have shifted their attention to methods to optimize recovery and outcomes of survivors of critical illnesses (1).

Inadequately treated pain, deep sedation, delirium, and reduced mobilization have emerged as risk factors for acute muscle wasting and weakness and persisting physical and cognitive dysfunction. The ABCDEF bundle represents an evidence-based guide for clinicians to focus their attention on some fundamental elements that dramatically impact patient recovery and outcomes. The ABCDEF bundle aim to improve and optimize pain management and choice of sedation, reduce delirium, duration of mechanical ventilation, and ICU-acquired weakness and sleep disruption, and promote greater ICU patient and family involvement in the care process (2, 3). Multiple studies demonstrated that the use of ABCDEF bundle improves ICU patient care and outcomes and reduces healthcare costs (4). The bundle has individual components that are clearly defined and help empower a multidisciplinary approach that includes the clinicians and the families on a broader perspective of shared care of critical illness. Recently, the R element was added for Respiratory-drive-control (ABCDEF-R) to underline the challenges associated with the implementation of the bundle in patients with ARDS (5).

Several factors have been identified as barriers to ABCDEF bundle implementation and diffusion (5–7), including patient-related factors (intubation, clinical instability, communication barriers associated with sedation and/or delirium) (8, 9) and elements such as lack of resources and increased ICU providers' workload (6).

The COVID-19 pandemic has raised new challenges related to ethical, medical humanity, communication, psychological, patient safety, and clinical risk management issues (10–12).

This article series has broadened our thinking and highlighted key concepts about the importance and feasibility of early mobility and light sedation also in very ill critical patients.

Keng et al. conducted a retrospective study to investigate the association between post-rehabilitation functional status, weaning, and survival outcome in prolonged mechanical ventilation (PMV) patients. Functional status was measured by the de Morton Mobility Index (DEMMI). In patients with PMV, post-rehabilitation DEMMI was an important prognostic factor independently associated with weaning success, hospital survival, and 3-month survival after RCC discharge.

Yuan et al. found that the change of position, evaluated through electrical impedance tomography (EIT), may improve the ventilation distribution in the study patients.

Chen et al. investigated the frequency and characteristics of intensive care unit-acquired weakness (ICU-AW) in Extracorporeal membrane oxygenation (ECMO) patients. They found that the duration of mechanical ventilation, duration of deep sedation during ECMO operation, APACHE II, and lowest albumin level were independent predictors of ICU-AW in patients with ECMO support.

Lippi et al. summarized the available evidence in literature on the efficacy of rehabilitative strategies to improve the weaning process and reduce mechanical ventilation (MV) duration. Shorter duration of MV is now recognized as a key moment to prevent the onset of severe complications and to ensure sustainability in terms of health care cost reduction.

Gitti et al. highlighted the key concept of sedation in ICU patients. A “calm, comfortable, and collaborative” patient allows active cognitive stimulation, earlier liberation from the endotracheal tube, active mobilization, and improved interaction with the healthcare team and the family; all these topics are important patient-centered outcomes.

These works highlighted the importance of factors so far overlooked and underestimated in the management of critically ill patients, but which are fundamental to ensure the optimal long-term quality of life. The aim of the ABCDEF bundle is to shift the attention beyond the walls of the ICU, laying the foundation for patient health after discharge from the unit.

## Author contributions

MV and AM compiled the manuscript. PB and KK undertook final amendments and approved the final version. All authors contributed to the article and approved the submitted version.

## Conflict of interest

The authors declare that the research was conducted in the absence of any commercial or financial relationships that could be construed as a potential conflict of interest.

## Publisher's note

All claims expressed in this article are solely those of the authors and do not necessarily represent those of their affiliated organizations, or those of the publisher, the editors and the reviewers. Any product that may be evaluated in this article, or claim that may be made by its manufacturer, is not guaranteed or endorsed by the publisher.
